# Supplementary material for: Child Labor in Family Tobacco Farms in Southern Brazil: Occupational Exposure and Related Health Problems
Source: Int J Environ Res Public Health. 2021 Nov 22;18(22):12255. doi: 10.3390/ijerph182212255 (PMC8620869; doi:10.3390/ijerph182212255)
Supplement: Supplementary file 1 [file ijerph-18-12255-s001.zip › ijerph-1446872-supplementary.pdf]

**Child Labor in Family Tobacco Farms in Southern Brazil: Occupational Exposure and Related Health Problems**

**Anaclaudia Gastal Fassa[1*], Neice Muller Xavier Faria[1], Ana Laura Sica Cruzeiro Szortyka[2], Rodrigo Dalke Meucci[3], Nadia Spada Fiori[1] and Maitê Peres de Carvalho[1]**

1 Department of Social Medicine, Faculty of Medicine, Federal University of Pelotas, Pelotas 96030-000, Brazil; neicef@yahoo.com.br (N.M.X.F.); nsfiori@yahoo.com.br (N.S.F.); maite_carvalho@yahoo.com.br (M.P.C.)
2 Psychology Course, Faculty of Medicine, Federal University of Pelotas, Pelotas 96030-000, Brazil; alcruzeiro@gmail.com
3 Faculty of Medicine, Federal University of Rio Grande, Rio Grande 96203-900, Brazil; rodrigodalke@gmail.com
* Correspondence: anaclaudia.fassa@gmail.com

## Supplementary File S1
## Individual Questionnaire - Selected Questions – Characterization of Health Problems

**Now let's talk about symptoms related to contact with green tobacco leaf (harvesting and handling). After picking tobacco, some people often experience dizziness or headache along with nausea or vomiting:**

Have you ever had these symptoms in your life right after picking tobacco?
(0) No (1) Yes (9) Ign

How many times?
___ ___ times (77) Many times (99) Ign

In the last month, have you had dizziness or headache along with nausea or vomiting after picking tobacco?
(0) No (1) Yes (9) Ign

In the past week, have you had or are you still experiencing dizziness or headache, as well as nausea or vomiting, after picking tobacco?
(0) No (1) Yes (9) Ign

**Now let's talk about pesticide poisoning.**

Have you ever had pesticide poisoning in your life?
(0) No, I have never had pesticide poisoning
(1) I had symptoms related to the use of pesticides, but not sure if it was poisoning
(2) Yes, I already had pesticide poisoning for sure
* The article considered that respondents had pesticide poisoning when they reported certainty in relation to it, alternative (2).

**Now answer some questions about your breathing and your lungs.**

Do you usually have a cough without having a cold?
(0) No (1) Yes (9) Ign

Have you ever had wheezing in your chest in the last 12 months?
(0) No (1) Yes (9) Ign

**Now let's talk about back pain. Consider the figure below to answer the questions.**

In the last 12 months, have you had back pain?
(0) No (1) Yes (9) Ign

If yes, ask if the respondent had pain in each of the regions indicating the location by color in the figure.

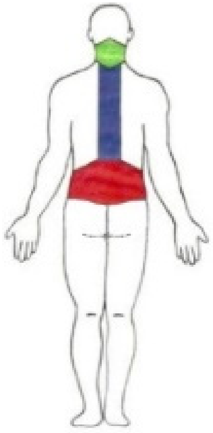

Cervical region (Neck – green color)
(0) No (1) Yes (9) Ign

Thoracic region (blue color)
(0) No (1) Yes (9) Ign

Low back region (red color)
(0) No (1) Yes (9) Ign
